# LncRNA LOC105369504 inhibits tumor proliferation and metastasis in colorectal cancer by regulating PSPC1

**DOI:** 10.1038/s41420-023-01384-3

**Published:** 2023-03-10

**Authors:** Ting Zhan, Xueting Cheng, Qingxi Zhu, Zheng Han, Kejing Zhu, Jie Tan, Men Liu, Wei Chen, Xiaoli Chen, Xiaohong Chen, Xia Tian, Xiaodong Huang

**Affiliations:** 1 Department of Gastroenterology, WuHan Third Hospital (Tongren hospital of WuHan University), 430060 Wuhan, China; 2 Department of Pharmacy, WuHan Third Hospital (Tongren hospital of WuHan University), 430060 Wuhan, China

**Keywords:** Cancer prevention, Mechanisms of disease

## Abstract

There is growing evidence that long non-coding RNAs (lncRNAs) are significant contributors to the epigenetic mechanisms implicated in the emergence, progression and metastasis of the colorectal cancer (CRC), but many remain underexplored. A novel lncRNA LOC105369504, was identified to be a potential functional lncRNA by microarray analysis. In CRC, the expression of LOC105369504 was markedly decreased and resulted in distinct variations in proliferation, invasion, migration and epithelial-mesenchymal transition (EMT) in vivo and in vitro. This study showed that LOC105369504 bound to the protein of paraspeckles compound 1 (PSPC1) directly and regulated its stability using the ubiquitin-proteasome pathway in CRC cells. The suppression of CRC by LOC105369504 could be reversed through PSPC1 overexpression.This study showed that in CRC, LOC105369504 was under-regulated and as a novel lncRNA, LOC105369504 exerted tumor suppressive activity to suppress the proliferation together with metastasis in CRC cells through the regulation of PSPC1. These results offer new perspectives on the lncRNA effect on the progression of CRC.

## Background

Colorectal cancer (CRC) is the third most prevalent malignant tumor affecting humans worldwide, as well as a significant cause of mortality [1]. Like numerous other types of cancer, CRC progression is a multi-stage process, including an accumulation of epigenetic genetic changes [2]. Genetic and molecular variations have a significant effect on these events and offer potential targets for treatment [3], so understanding the underlying mechanism behind CRC progression is important.

LncRNAs are a type of multifunctional non-coding RNAs, which exceeds two hundred nucleotides in size and lacks the potential for coding [4]. They were previously regarded as non-functional fragments produced during transcription, but many recently published research reports have suggested that lncRNAs exert an essential effect in bio-processes, including influencing the occurrence and development of multiple cancers such as gastric cancer, breast cancer, prostate cancer, lung cancer, as well as CRC [5].

Numerous abnormal lncRNAs have been recently detected in CRC. These abnormally expressed lncRNAs were viewed as specific biomarkers for diagnosis, prognosis and assessment and treatment [6], and have been confirmed to influence the biological processes of CRC such as drug resistance, metastasis and proliferation [7–9]. There is a competitive combination between lncRNA LUNAR1 and mir-495-3p, which increases the expression of the oncogene, MYCBP, and facilitates CRC invasion, migration and proliferation [10]. Zhou et al. [11] found that a hypoxia-induced antisense lncRNA STEAP3-AS1 that was highly expressed in clinical CRC tissues and positively correlated with poor prognosis of CRC patients. It has also been shown lncRNA WASH5P can suppress CRC and be a potential candidate therapeutic target via the phosphatidlinositol 3-kinase /protein kinase B (PI3K/AKT) signaling [12].

This study examined lncRNA expression profiles in samples of CRC combined with corresponding paracancerous tissues, utilizing transcriptome microarray technology. The role of abnormally expressed lncRNA in CRC was evaluated and LOC105369504, a novel lncRNA that remains unreported in literature, was selected as the object of research. Expression changes, biological functions and the mechanistic role of LOC105369504 in CRC were defined and the outcomes suggested that it suppressed CRC proliferation and metastasis by promoting paraspeckle component 1 (PSPC1) protein degradation.

## Results

### Differential lncRNA expression profiles and LOC105369504 downregulation in CRC

**Fig. 1:**
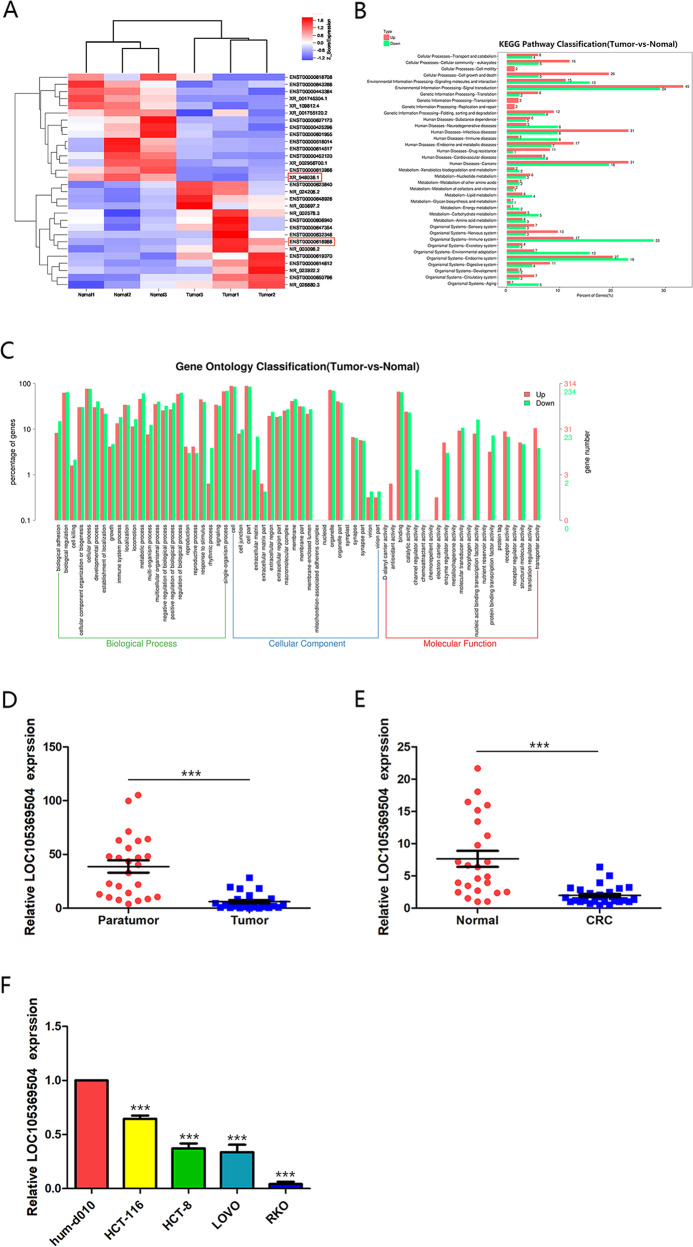
Differential lncRNA expression profile and LOC105369504 is downregulated in CRC. **A** Heat map of the top 30 differentially expressed lncRNAs between CRC and paracancerous tissues. **B** KEGG enrichments of the top 30 DEGs. **C** GO enrichments of the top 30 DEGs. **D** Expression of LOC105369504 was decreased in CRC tissues compared to paracancerous tissues. **E** LOC105369504 expression was reduced in plasma of patients with CRC compared to normal controls. **F** Expression of LOC105369504 was attenuated in CRC cells compared to normal colorectal epithelial cells. All data are described as the mean ± SD. ***p < 0.001.

With the aim of exploring the effect of lncRNAs in CRC progression, the differentially expressed lncRNAs in CRC and the adjacent tissues were first screened. Based on the lncRNAs microarray test shown in Fig. 1A, there were 896 differentially expressed lncRNAs, including 547 upregulated lncRNAs and 349 downregulated lncRNAs in CRC tissues. Thirty significant differentially expressed genes (DEGs) were chosen for gene enrichment analysis. The enrichments of Kyoto encyclopedia of genes and genomes (KEGG) and gene ontology (GO) reflected that DEGs were associated with carcinogenic pathways and processes, as shown in Fig. 1B, C.

The top two DEGs, lncRNA AL161431.1, with a transcript name of enst00000618966 and lncRNA LOC105369504 whose transcript name was xr_948036.1, were chosen for in-depth research on eight pairs of CRC and paracancerous tissues using qRT-PCR. The outcomes revealed that only LOC105369504 was highly consistent with the lncRNAs microarray analysis, but AL161431.1 was inconsistent with the lncRNAs microarray analysis, as shown in Supplementary Fig. 1. Thus, LOC105369504 was chosen for further experimentation.

The expression of LOC105369504 in 24 paired CRC and paracancerous tissues was then determined and it was shown that it was decreased in CRC tissues compared to the paracancerous tissues, as shown in Fig. 1D. The outcomes of serum samples were consistent with those of tissue samples in Fig. 1E. As displayed in Fig. 1F, the expression of LOC105369504 in cell lines was determined at the same time. The expression of LOC105369504 in HUM-d010, the normal cell line of colorectal epithelial tissues was higher than those in the CRC cells, HCT-116, HCT-8, LOVO and RKO. The above data agreed with the microarray results and indicated that LOC105369504 might participate in the emergence and progression of CRC.

### LOC105369504 inhibited CRC cell proliferation and metastasis mediated by EMT in vitro

**Fig. 2:**
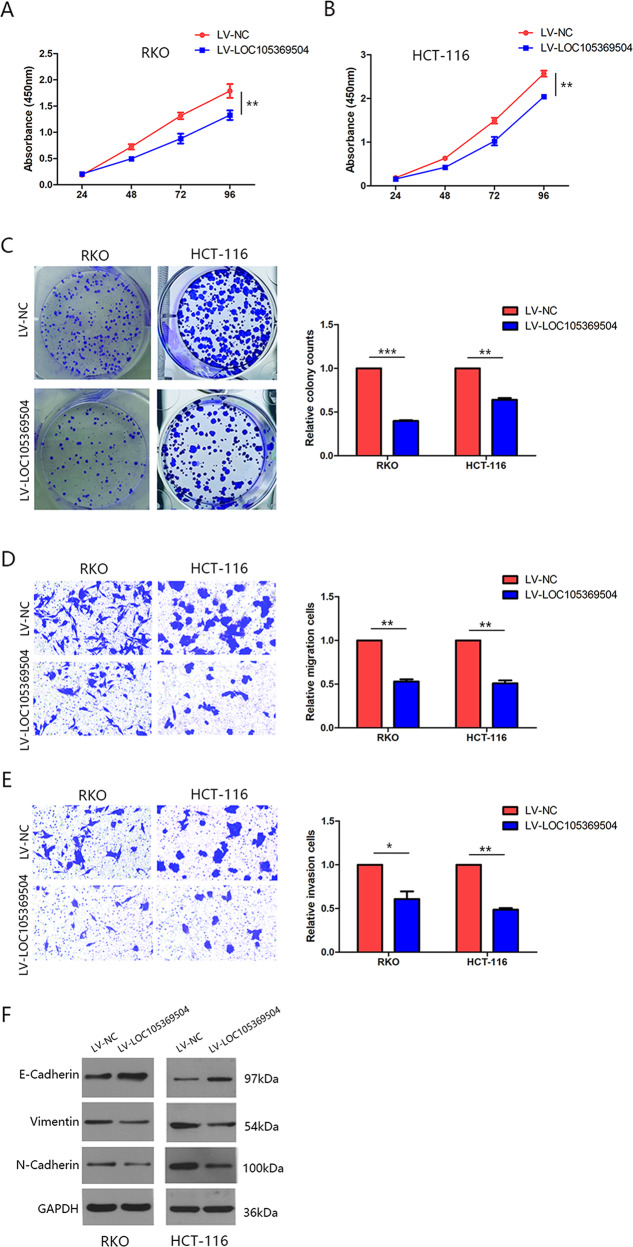
LOC105369504 suppresses CRC cell proliferation and metastasis mediated with EMT in vitro. **A**, **B** The effect of LOC105369504 on proliferation in RKO and HCT-116 cells transfected by LV-NC or LV-LOC105369504 through the CCK-8 assay. **C** Colony formation analyses for CRC cells after LOC105369504 was overexpressed. **D**, **E** Transwell analysis was applied for determining the CRC migration together with invasion after LOC105369504 was overexpressed. **F** Western blotting indicated increased epithelial marker expression and attenuated mesenchymal marker expression in the CRC cells transfected with LV-LOC105369504. All the data are expressed as the mean ± SD. *p < 0.05, **p < 0.01, ***p < 0.001.

Some functional tests were used to better understand the effects of LOC105369504 on CRC progression. Since the relative expression of LOC105369504 in RKO cells was the lowest and that of HCT-116 was the highest, the LOC105369504 expression in HCT-116 and RKO cells was over expressed with LV- LOC105369504. The transfection efficiency in RKO and HCT-116 cells were verified using qRT-PCR (Supplementary Fig. 2). The curve of cell proliferation identified through CCK-8 analysis suggested that the upregulation of LOC105369504 attenuated the proliferation of cells, as shown in Fig. 2A and B. Colony formation assays indicated that LOC105369504 overexpression could attenuate the number of colonies in Fig. 2C.

LOC105369504 overexpression decreased the migration and invasion of RKO and HCT-116 cells (Figs. 2D, E).

Epithelial-mesenchymal transition (EMT) is a key stage in the process of CRC metastasis [13], which is applied to detect whether LOC105369504 suppressed metastasis mediated by EMT.E-cadherin was employed as the epithelial marker of CRC cells and vimentin and N-cadherin served as mesenchymal markers in western blots. As displayed in Fig. 2F, LOC105369504 overexpression increased E-cadherin expression, but attenuated vimentin and N-cadherin expression. These findings supported the discovery that LOC105369504 inhibited the proliferation of CRC cells and the metastasis mediated by EMT in vitro.

### LOC105369504 directly interacted with PSPC1, decreasing its stability and expression

**Fig. 3:**
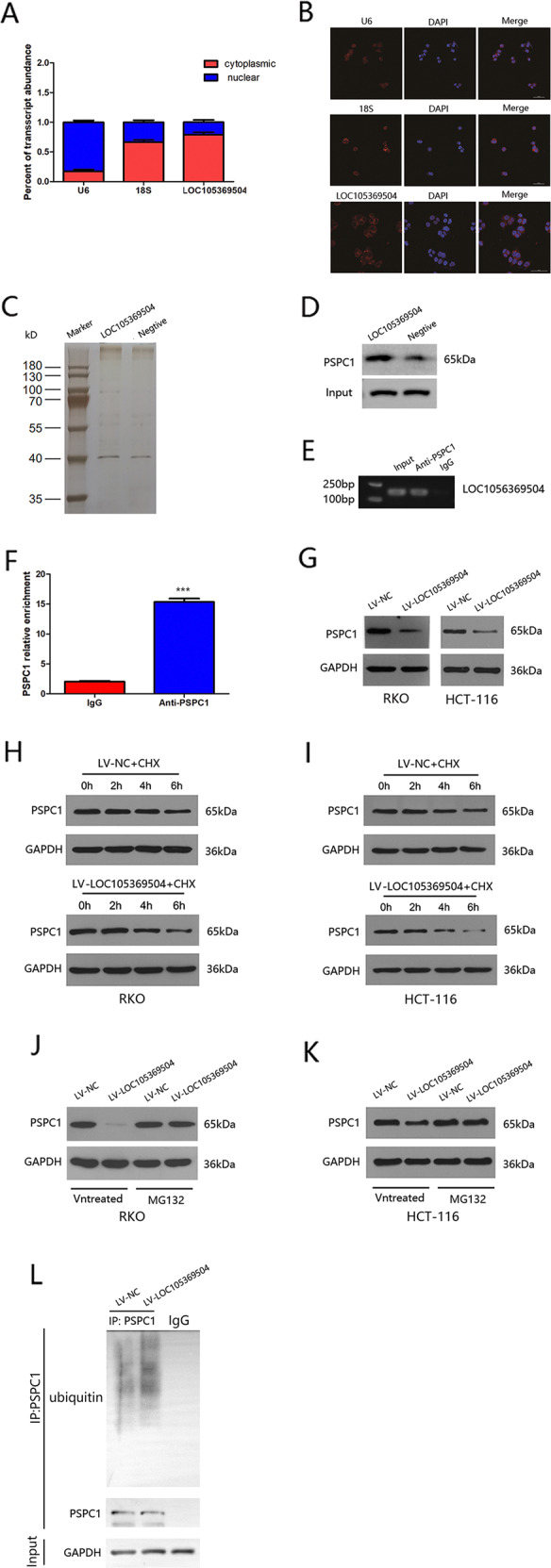
LOC105369504 decreases the stability of PSPC1 by the ubiquitin-proteasome pathway. **A** Cytoplasmic and nuclear RNA fractions were separated from RKO cells and the results showed that LOC105369504 was located in the cytoplasm. **B** FISH was implemented for further confirming that LOC105369504 was located in the cytoplasm. **C**, **D** RNA pull-down assays after silver staining and western blotting to identify the protein expression of PSPC1 in RKO cells. **E**, **F** RIP assay for PSPC1 followed by agarose gel electrophoresis and qRT-PCR suggested that LOC105369504 could bind PSPC1 protein. **G** In CRC cells, the PSPC1 expression with or without overexpression of LOC105369504 was identified by western blotting. **H**, **I** Western blotting showing the protein level of PSPC1 after treating the cells with CHX (50 μg/mL). **J**, **K** Western blotting displaying the protein level of PSPC1 after treating cells with or without MG132 (10 μmol/mMl). **L** Western blot analysis of ubiquitinated PSPC1 immunoprecipitated from RKO cells with or without overexpression of LOC105369504. MG132 (10 μmol/Ml) was utilized to treat cells to suppress the proteasome. All the data are described as mean ± SD. ***p < 0.001.

The location of lncRNAs provide insight into their underlying interactive partners and their cellular effect [14]. Subcellular fractionation assays and FISH analysis found that LOC105369504 was in the cytoplasm of RKO cells (Fig. 3A, B). An earlier research [15] confirmed that there was an interaction between lncRNAs and proteins to regulate the expression of genes, so the proteins that may interact with LOC105369504 were further explored. RNA pull-down assays followed by MS analysis (Table S2) showed that PSPC1 could bind to LOC105369504 specifically, as shown in Fig. 3C. An immunoprecipitation assay further demonstrated that PSPC1 bound to LOC105369504 directly (Fig. 3D). In RKO cells, there was an interaction between PSPC1 and LOC105369504, which was confirmed by agarose gel electrophoresis and RIP assays in Fig. 3E, F.

Cytoplasmic lncRNAs are responsible for post-transcriptional regulation of protein modifications [16]. The overexpression of LOC105369504 resulted in a decrease in the protein expression of PSPC1 in RKO and HCT-116 cells (Fig. 3G). Cycloheximide (CHX), a protein synthesis suppressor, was used to observe the LOC105369504 effect against the degradation of PSPC1 and the results of western blotting displayed in Fig. 3H, I showed that overexpression of LOC105369504 reduced PSPC1, thus, indicating that it could regulate PSPC1 through protein degradation. Given that the degradation of protein is mediated by the ubiquitin-proteasome pathway [17], LV-LOC105369504 cells were treated with a proteasome inhibitor MG132, which markedly restored the protein levels of PSPC1, as shown in Fig. 3J, K. Those findings suggested that the ubiquitin-proteasome pathway participated in the degradation of PSPC1 induced by LOC105369504. To further validate this finding, the poly-ubiquitination of PSPC1 in RKO cells transfected with LV-LOC105369504 was evaluated and was significantly increased in Fig. 3L. These data showed that LOC105369504 bound PSPC1 directly and attenuated its stability using the ubiquitin-proteasome pathway in CRC cells.

### LOC105369504 inhibited tumor proliferation and metastasis in a PSPC1-mediated mechanism

**Fig. 4:**
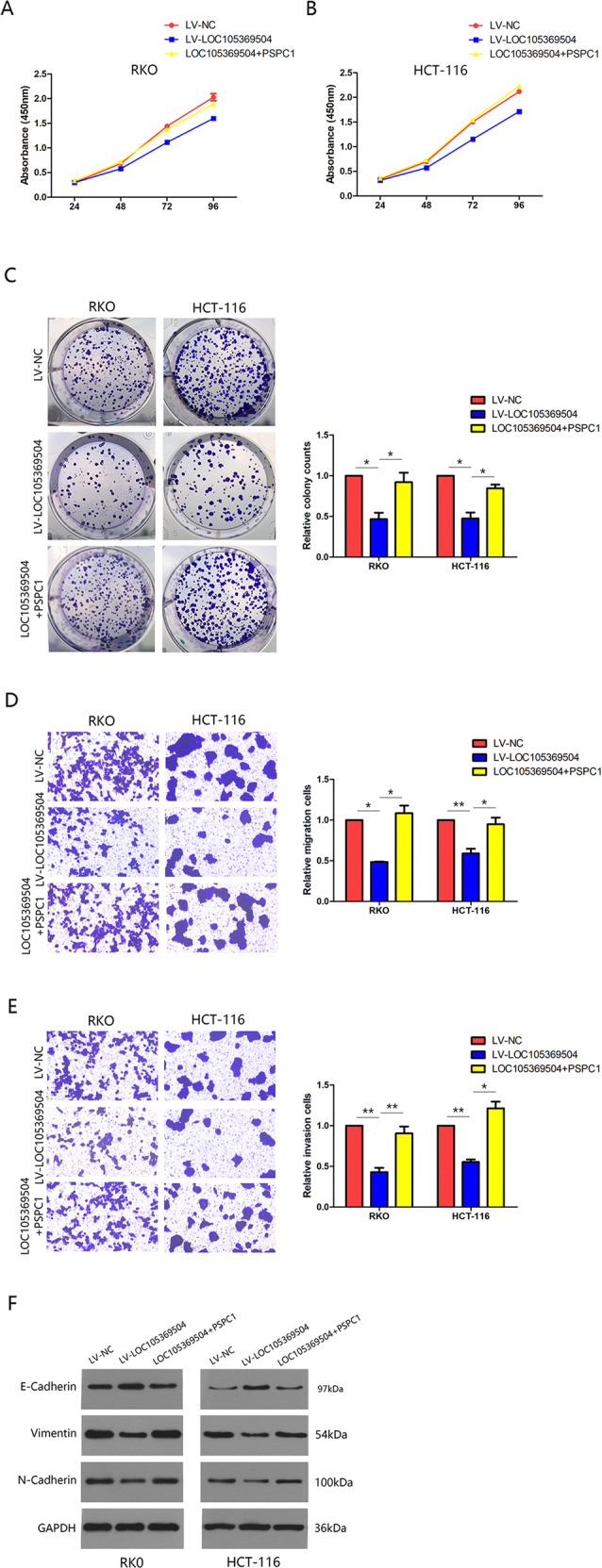
LOC105369504 suppressed tumor proliferation and metastasis in a PSPC1-mediated manner. **A**, **B** The cell proliferation of RKO and HCT-116 cells transfected with LV-LOC105369504 or LV-LOC105369504 plus the PSPC1 expression vector. **C** Colony formation assays for CRC cells transfected with LV-LOC105369504 or LV-LOC105369504 plus the PSPC1 expression vector. **D**, **E** Transwell assay for CRC cells transfected with LV-LOC105369504 or LV-LOC105369504 plus the PSPC1 expression vector. **F** The EMT-associated genes were identified through western blotting in CRC cells transfected with LV-LOC105369504 or LV-LOC105369504 plus the PSPC1 expression vector. All data are expressed as the mean ± SD. *p < 0.05, **p < 0.01.

To explore whether LOC105369504 played a role in CRC progression by regulating PSPC1, a series of rescue tests with CRC cells transfected with LV- LOC105369504 or LV- LOC105369504 plus PSPC1 expression vector were conducted. The outcomes of colony formation and CCK-8 assays indicated that LOC105369504 inhibit the cell proliferation in CRC cells and PSPC1 overexpression inversed LOC105369504 suppression to endogenous PSPC1 as seen in Fig. 4A–C. The overexpression of LOC105369504 similarly suppressed migration, invasion and EMT in CRC cells, but PSPC1 overexpression reversed the anti-cancer effect of LOC105369504, as seen in Fig. 4D–F. Those findings confirmed that LOC105369504 inhibited the proliferation of tumor formation and metastasis mediated by EMT in a PSPC1-mediated mechanism.

### LOC105369504 functions in vivo

**Fig. 5:**
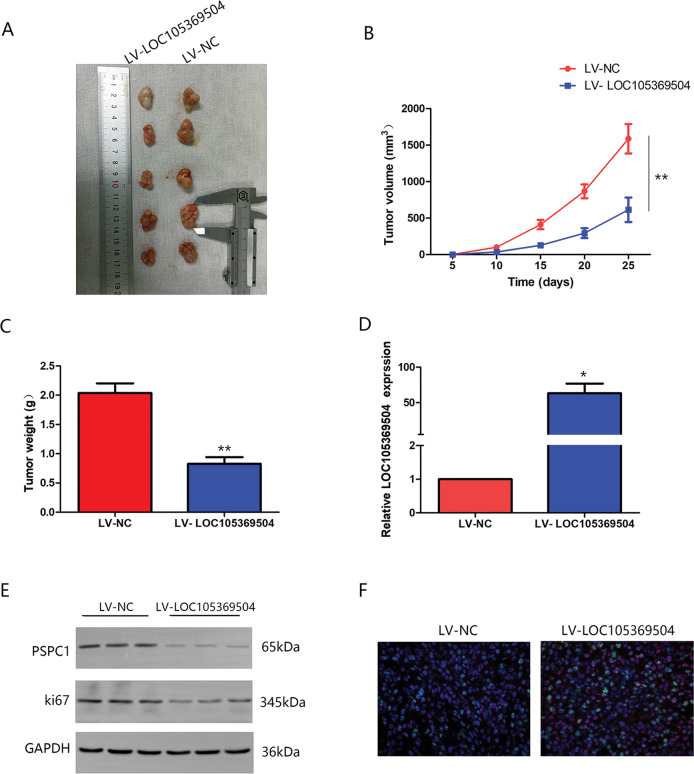
LOC105369504 functions in vivo. **A**–**C** LOC105369504 markedly suppressed the xenograft growth generated by the RKO cells compared to the NC group. **A** Representative images of tumors separated from tumor-bearing mice. **B** Tumor volume of nude mice. **C** Tumor weight of nude mice. There are five mice in each group (*n* = 5). **D** LOC105369504 expression was determined by qPCR. **E** Western blotting was applied to determine the expression of Ki67 and PSPC1. **F** Expression of PSPC1 (red light) and Ki67 (green light) was detected by immunofluorescence. All data are described as the mean ± SD. *p < 0.05, **p < 0.01.

To examine the regulatory effects of LOC105369504 on CRC in vivo, RKO-LV-NC and RKO-LV- LOC105369504 cells were injected subcutaneously into nude mice. In accordance with this study’s in vitro results, the tumor volumes and weights generated through RKO-LV-LOC cells were significantly smaller than those generated by control cells, as seen in Fig. 5A–C. The qRT-PCR results showed (Fig. 5D) that LOC105369504 expression in the overexpression group was increased compared to the NC group. The western blotting results (Fig. 5E) showed that the group of LOC105369504 overexpression had lower Ki-67 and PSPC1 expression compared with NC group, while the results of IF analysis (Fig. 5F) were consistent with those of western blotting assays. These findings indicated that LOC105369504 might suppress CRC growth in vivo through PSPC1.

## Discussion

Numerous studies have shown that 98% of the transcripts in the human genome are non-coding RNAs (ncRNAs) [18, 19], among which lncRNAs are transcripts exceeding 200 nucleotides and possess no protein coding potential [20]. Accumulating evidence has demonstrated that lncRNAs exert a crucial effect in all phases of carcinogenesis and tumor progression [17, 21, 22]. At present, a lot of lncRNAs have been reported to be abnormally expressed in CRC and some of these lncRNAs are upregulated in tumors and act as oncogenes, while others are downregulated and serve as tumor suppressors [23]. As significant contributors of epigenetic mechanisms, lncRNAs participate in CRC occurrence, development and metastasis [24–26]. Thus, studying the differentially expressed lncRNAs in CRC can not only help to understand the progress of CRC from the molecular mechanism, but also help to find new molecular markers and therapeutic targets.

As an initially transcribed RNA or a mature spliced RNA, LncRNAs can regulate the expression of significant genes at multiple levels through epigenetic regulation and by modulating transcription, post-transcriptional processes, translation, and protein modification [27]. It is reported that interaction of lncRNA MIR100HG with hnRNPA2B1 facilitates m6A-dependent stabilization of TCF7L2 mRNA and colorectal cancer progression [28]. LncRNA ITGB8-AS1, as a competitive endogenous RNA, facilitates the growth and migration of CRC via the focal adhesion signaling mediated with integrin [9].LncRNA CCDC144NL-AS1 facilitates the proliferation of CRC by regulating the miR-363-3p/GALNT7 axis [29] and LncRNA GAS6-AS1 facilitates tumorigenesis and metastasis of CRC by regulating TRIM14 through miR-370-3p/miR-1296-5p [30]. Nuclear lncRNAs can interact with chromatin, and modulate gene expression and the processing of RNA at the transcriptional level. Cytoplasmic lncRNAs can influence mRNA stability and translation, and regulate the cellular signaling pathways [16].

In this study, subcellular fractionation and FISH assays revealed that LOC105369504 preferentially localized to the cytoplasm, showing that it may exert an effect in regulating signal transduction, translation or post transcriptional modulation by regulating mRNA stability or interacting with proteins or miRNAs [14, 31]. To further understand the potential molecular mechanisms involved, RNA pulldown and mass spectrum assays were performed, and PSPC1 was shown to be an interactive protein of LOC105369504, which reduced the stability of PSPC1 and promoted its degradation by the ubiquitin-proteasome pathway in CRC.

PSPC1 was first shown to be a unique type of nuclear structural protein, known as paraspeckle [32]. Studies in recent years have demonstrated that PSPC1 was overexpressed in liver cancer, nasopharyngeal cancer, lung cancer and breast cancer, which is a negative prognostic factor for patient survival [33–35]. Recent research indicates that PSPC1 is a new contextual determinant of aberrant subcellular translocation of oncogenes in the growth and metastasis of cancer cells and modulated genes associated with cancer stem cells and EMT [36]. PSPC1 is an interaction partner of Smad2/3 as TGF-β1 the contextual determinants of the response will be dichotomous TGF-β1 function changes from tumor inhibition in precancerous cells to metastasis promotion in malignant cells [33, 37]. Moreover, PSPC1, as a substrate of nuclear PTK6, is the contextual determinant of PTK6 nucleocytoplasmic shuttling and modulates the switch of tumor-suppressive PTK6 in the nucleus of normal [38].Through a series of rescue experiments, it was confirmed that as a novel lncRNA, LOC105369504 exerted tumor suppressive activity to inhibit cell proliferation and metastasis in CRC by regulating PSPC1.

## Conclusion

This work demonstrated that the expression of LOC105369504 was attenuated in CRC and suppressed its proliferation, metastasis and tumorigenesis in vivo and in vitro. The molecular mechanism by which LOC105369504 functioned as an anti-oncogene in CRC was defined and these results give novel insights into the effect of lncRNA in CRC progression. Accompanied with further investigations, these outcomes may offer therapeutic targets for advanced CRC.

## Materials and methods

### Clinical specimens

The 24 pairs of CRC together with the associated adjacent tissues used in this paper were gathered from patients who received surgical resection but did not undergo the preoperative chemotherapy and radiotherapy in Tongren Hospital of Wuhan University. These samples were immediately rapidly frozen in the liquid nitrogen and maintained under a temperature of −80 °C until RNA was extracted. From Tongren Hospital of Wuhan University, the plasma samples from 34 patients with CRC and thirty healthy controls could be collected. In the EDTA tubes, all the peripheral plasma samples were gathered and treated within 4 h through centrifugation under a temperature of 4 °C at 1000 *g* for 15 min. The plasma was subsequently gently transferred to a fresh EP tube (1.5 ml) without RNase/DNase (Axygen, Union City, CA) and placed under a temperature of −80 °C. The patients who participated in this work expressed informed consent. This research was authorized through the Ethics Committee of Tongren Hospital of Wuhan University.

### Cell lines and cell culture

The cell lines HCT8, HCT-116, LOVO and RKO applied in the research were provided via the American Type Culture Collection (ATCC, Virginia, USA). From iCell Bioscience Inc (Shanghai, China), the normal human colon epithelial cells (HUM-d010) were acquired. In Ham’s F12K medium (Thermo Fisher Scientific, USA), the LOVO and HCT-116 cells were inoculated. While the HT-29 cell was inoculated in the RPMI-1640 medium (Thermo Fisher Scientific, USA). RKO was inoculated in MEM with non-essential amino acid medium (Shanghai BasalMedia, China) containing 1% sodium pyruvate (Shanghai BasalMedia, China) .HUM-d010 was cultured in Special medium for primary colonic epithelial cells (Shanghai saibaikang, China). All media were supplemented with cyanine streptomycin (1%, Thermo Fisher Scientific, USA) and FBS (10%, NQBB, USA). In the humidified incubator, the cells were cultivated under a temperature of 37 °C with 5% CO2.

### Microarray and bioinformatics analyses

The total RNA from six tissues, containing three primary tissues of CRC together with three paracancerous tissues, were extracted through Trizol reagent (Invitrogen, Carlsbad, CA, USA), and it was next purified through RNeasy Mini Kit (Qiagen, Valencia, CA, USA). Subsequently, the third company (CapitalBio Corporation, China) supposed to utilize Agilent 4 × 44 Kv2 expression arrays (Agilent Technologies, Inc.) for exploring the gene expression profile of RNA. The DEGs were counted through R program and next clustered. Limma package was applied for determining DEGs. P1.5 is regarded statistically significant. Kyoto Encyclopedia of Genes and Genomes (KEGG) and Gene Ontology (GO) conduct enrichment analysis of DEGs through utilizing the Database for Annotation, Visualization as well as Comprehensive Discovery.

### Quantitative real-time polymerase chain reaction (qRT-PCR)

With Trizol reagent (Invitrogen, California, USA), the total RNA was separated from tissues and cell lines of CRC. Complementary DNA (cDNA) was reverse transcribed (RT) with the PrimeScript Reverse Transcriptase Reagent Kit (TakaRa, Tokyo, Japan). SYBR Green PCR Master Mix (TaKaRa, Tokyo, Japan) was applied for amplifying the cDNA fragments. GAPDH acts as the endogenous control. Table S1 lists the sequences of the positive and antisense primers applied. All the outcomes are counted and described as 2 − ΔΔCT.

### Cell transfection

Shanghai GeneChem (Shanghai, China) provides LOC105369504 overexpression lentivirus (LV-LOC105369504)、PSPC1 overexpression lentivirus (LV-PSPC1) and control lentivirus vector(LV-NC). In accordance with the instructions of manufacturer, the lentivirus was transfected.

### CCK8 assay

Cell proliferation was identified with CCK-8 (Dojindo, Japan). Transfected cells were inoculated on plates (96 well, 4 × 10^3^ cells each well) for 1, 2, 3 and 4 days. Add the CCK-8 solution (10 mL) to each well, cultivated under a temperature of 37 °C for another 2 h, and at 450 nm, EnSpire Multimode Plate Reader (PerkinElmer, USA) was employed for identifying the absorbance.

### Colony formation assay

The cells were inoculated in plates (6 well) at 400 cells/well density; After the cultivation in 37 °C complete medium for 2 weeks, visible cell colonies occurred. The methanol was fixed with colonies for 15 min and stained through crystal violet (0.1%) for 30 min.

### Transwell migration and invasion assays

Based on the instructions of manufacturer, Transwell chambers (8 µm pore size; Costar) were employed for the test of cell migration, and Matrigel (BD Biosciences, San Jose, CA, United States) together with transwell chambers (with a pore size of 8 µm; Costar) were applied for Transwell invasion test. For the analysis of invasion and migration, the medium involving FBS (20%) in lower chamber was applied as a chemical attractant. After 1 day, non-invasive or non-migrating cells were removed from the upper filter surface employing cotton swabs. The invasive and migrating cells situated at the chamber lower side were fixed with paraformaldehyde (4%), which were stained by crystal violet for half an hour. Subsequently, the cells in the upper part of the chamber were wiped with cotton swab, and the cells existing in the chamber bottom were imaged and next counted under randomly five fields with microscope.

### Western blot analysis

Based on the instructions of manufacturer, Transwell chambers (8 µm pore size; Costar) were employed for the test of cell migration, and Matrigel (BD Biosciences, San Jose, CA, United States) together with transwell chambers (with a pore size of 8 µm; Costar) were applied for Transwell invasion test. For the analysis of invasion and migration, the medium involving FBS (20%) in lower chamber was applied as a chemical attractant. After 1 day, non-invasive or non-migrating cells were removed from the upper filter surface employing cotton swabs. The invasive and migrating cells situated at the chamber lower side were fixed with paraformaldehyde (4%), which were stained by crystal violet for half an hour. Subsequently, the cells in the upper part of the chamber were wiped with cotton swab, and the cells existing in the chamber bottom were imaged and next counted under randomly five fields with microscope.

### Immunoprecipitation (IP) assay

IP assays were conducted with anti-FLAG/DYKDDDDK Tag (Cell Signaling Technology, USA, 1–2 mg each test) together with anti-PSPC1 antibody (Abcam, USA, 1–2 mg each test), and anti-ubiquitin antibody (American Cell Signaling Technology, 1:1000) was utilized to identify the protein through Western blotting in accordance with the instructions of manufacturer.

### RNA pull-down, mass spectrometry and RNA immunoprecipitation assays

Magnetic RNA-Protein Pull-Down kit (Pierce, MA, USA) was employed for RNA pull-down following the instructions of manufacturer. The protein bands on gel were silver stained. The interest bands were determined and demonstrated through mass spectrometry (MS) together with Western blotting, respectively. While in accordance with the protocol of manufactures, RIP was implemented with Magna RIP RNA-Binding Protein Immunoprecipitation Kit (Millipore, MA, USA).

### Cytoplasmic and nuclear RNA fraction

Following the manufacturer’s instructions, utilizing the cytoplasmic and nuclear RNA Purification Kit (Norgen BioTek, Canada) to extract the nuclear and cytoplasmic RNA. RT-PCR was exploited for the evaluation of the lncRNA target expression in the nuclear/cytoplasmic part, and next ImageJ software was employed for quantifying the bands on gel image.

### Fluorescence in situ hybridization (FISH) assay

The construction of LOC105369504 FISH probe was implemented with Ribo Bio Technology (Guangzhou, China). The cells were firstly fixed in the paraformaldehyde (4%), subsequently infiltrated in PBS, and PBS was mixed with Triton X-100 (0.5%). The cells were next hybridized with RNA FISH probes labeled with Cy3 under a temperature of 37 °C. Afterwards, DAPI (Beyotime Biotechnology, shanghai,China) was employed for staining the cells. The confocal microscope was exploited to observe the existence of LOC105369504.

### Animal experiments

Beijing Vital River Laboratory Animal Technology (Beijing, China) provides the male BALB/c nude mice aged 6 weeks and raised under given pathogen free environments. Subcutaneously inject 5*106 RKO-LV- LOC105369504 and RKO-LV-NC cells. There were five animals in each of the treatment group. The volume of tumor was determined every 5 days, which was monitored through detecting the width (W) and length (L) with calipers, and counted through the below formula: tumor volume = L*W^2^/2. After 25 days, all of the mice were killed. For each tumor, its subcutaneous weight was detected and the tumor was applied for the in-depth exploration. This work meets the requirements of the animal experiment ethics committee of Tongren Hospital of Wuhan University.

### Immunohistofluorescence (IF) assay

The samples of tumor tissue were fixed with formalin, which were embedded in paraffin and cut into the thick sections (ranged from 4 to 5 mm), then boiled in citric acid solution (10 mM, with a pH of 6.0) for antigen recovery, and subsequently sealed in phosphate buffered saline involving normal serum of the species (10%) at RT for 60 min. After incubating with ki67 (1:150, abcam, USA) and PSPC1 (1:150, abcam, USA) specific antibodies, the fluorescent secondary antibodies were incubated with sections in darkness at RT for 120 min. Eventually, the slides were installed through the addition of DAPI-Fluoromount-G (Southern Biotech, SBA, Beijing, China). Fluorescence microscope was conducted to acquire the images.

### Statistical analysis

SPSS and GraphPad Prism were applied for statistical analyses and illustrated data, respectively. Each of the experiment were conducted for three or more times. One-way ANOVA or two-tailed Student’s *t* test were utilized for statistically analyzing the experimental data. Unless otherwise specified, the data were described as mean ± SD. The difference of *p* below 0.05 was viewed statistically significant.

## Supplementary information

Supplementary Figure 1

Supplementary Figure2

Table S1

Table S2

supplementary figure legends

Full and uncropped western blots

Full and uncropped western blots

Full and uncropped western blots

Full and uncropped western blots

Full and uncropped western blots

Full and uncropped western blots

Full and uncropped western blots

Full and uncropped western blots

Full and uncropped western blots

Full and uncropped western blots

Full and uncropped western blots

Full and uncropped western blots

## Data Availability

The data that support the findings of this study are openly available in figshare, 10.6084/m9.figshare.21137128.
