# Supplementary figures and images for: LncRNA LOC105369504 inhibits tumor proliferation and metastasis in colorectal cancer by regulating PSPC1
Source: Cell Death Discov. 2023 Mar 10;9:89. doi: 10.1038/s41420-023-01384-3 (PMC9998613; doi:10.1038/s41420-023-01384-3)

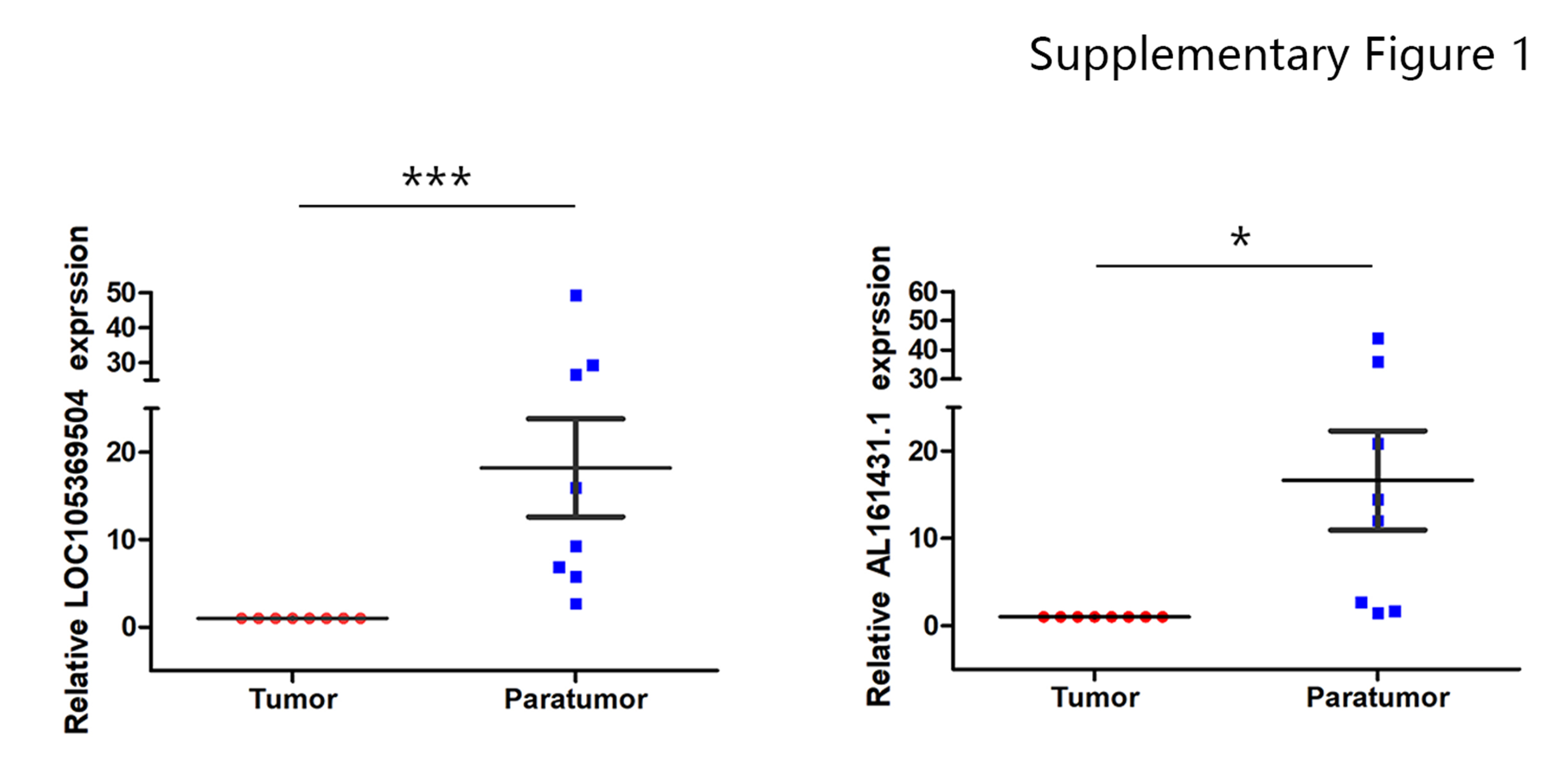

Supplement: Supplementary file 1 — Supplementary Figure 1 [file 41420_2023_1384_MOESM1_ESM.jpg]

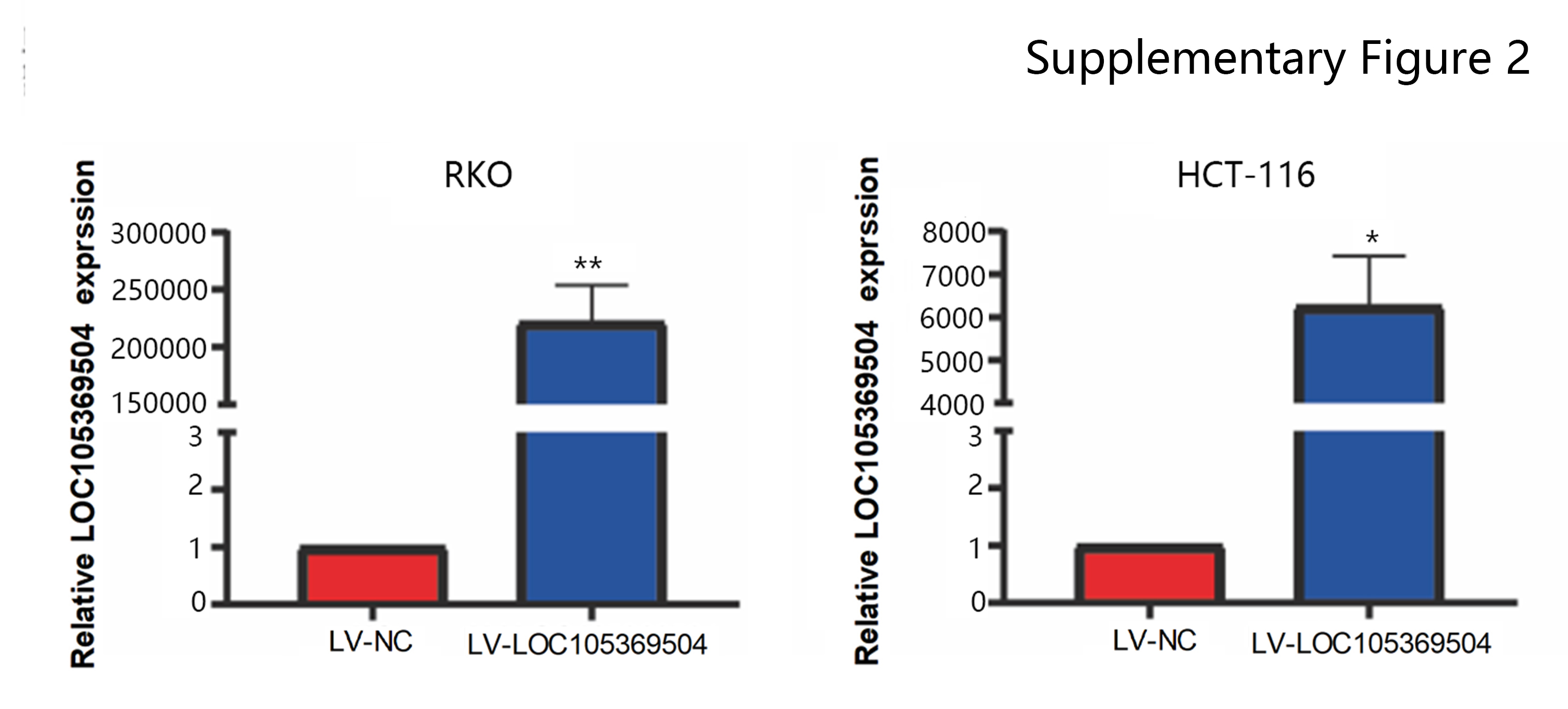

Supplement: Supplementary file 2 — Supplementary Figure2 [file 41420_2023_1384_MOESM2_ESM.jpg]

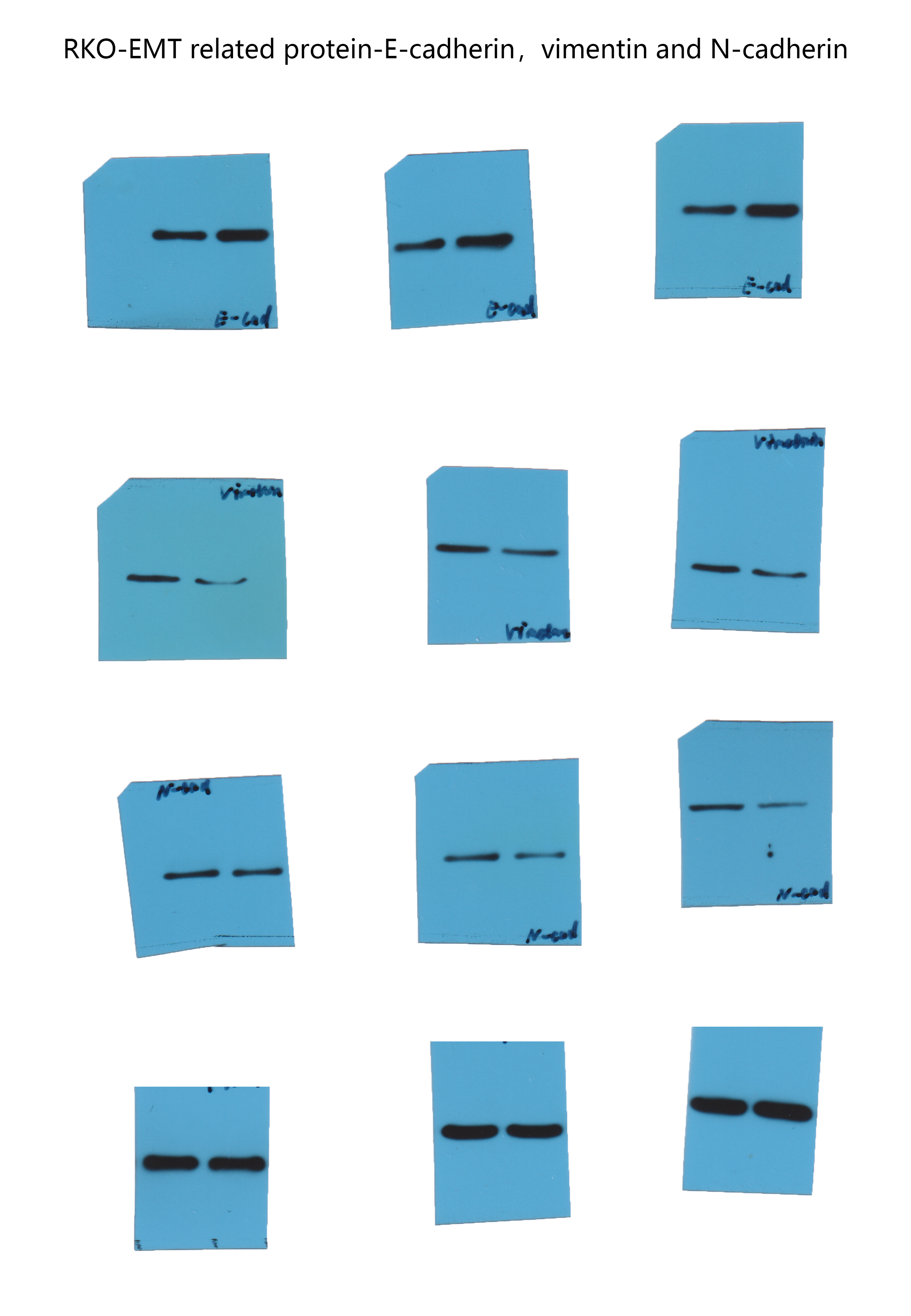

Supplement: Supplementary file 6 — Full and uncropped western blots [file 41420_2023_1384_MOESM6_ESM.tif]

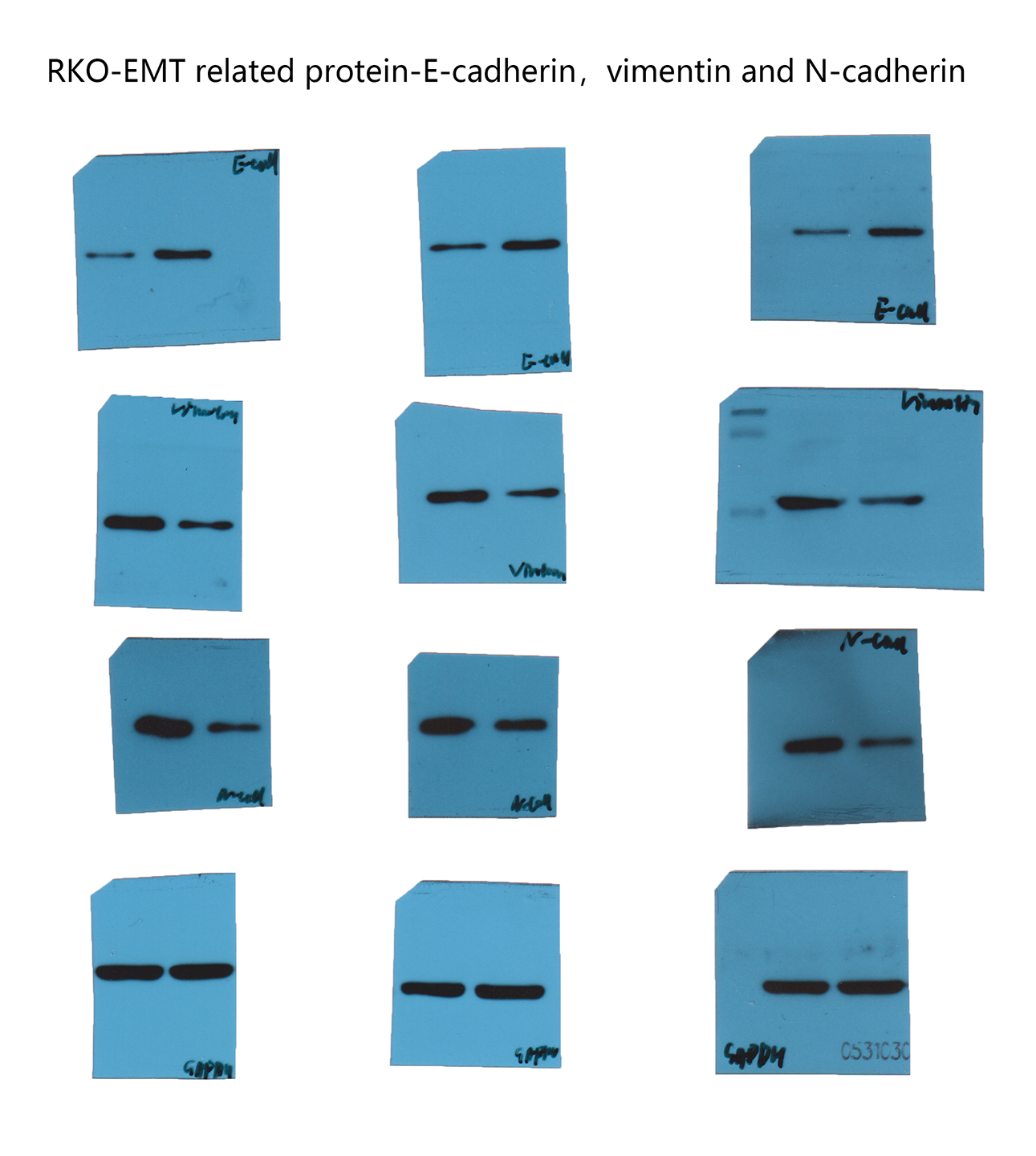

Supplement: Supplementary file 7 — Full and uncropped western blots [file 41420_2023_1384_MOESM7_ESM.tif]

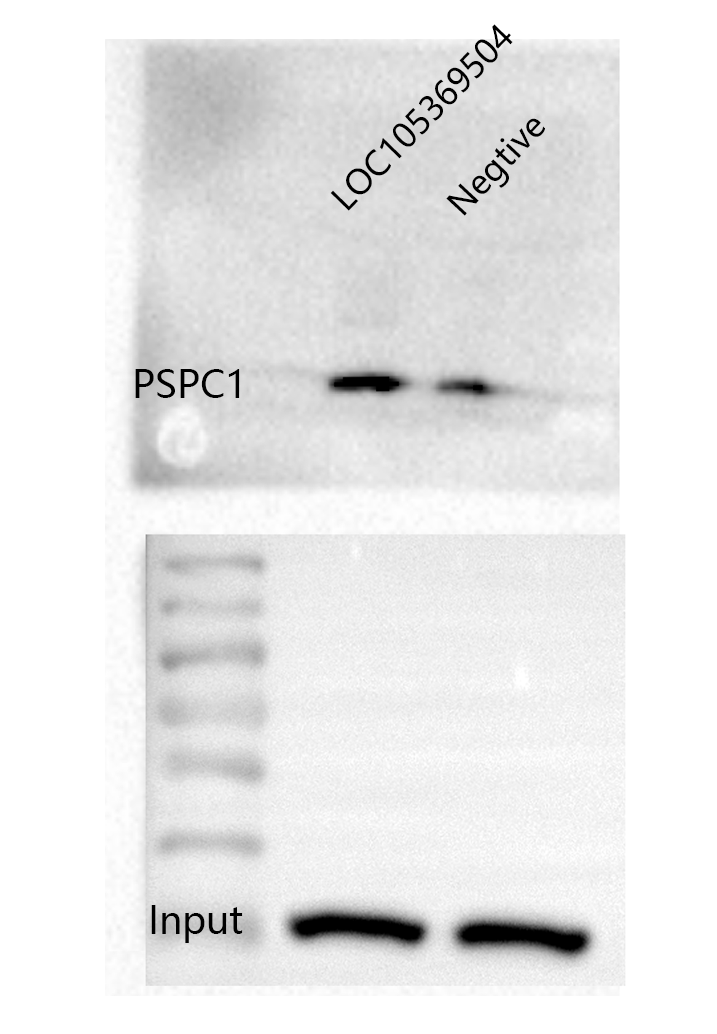

Supplement: Supplementary file 8 — Full and uncropped western blots [file 41420_2023_1384_MOESM8_ESM.tif]

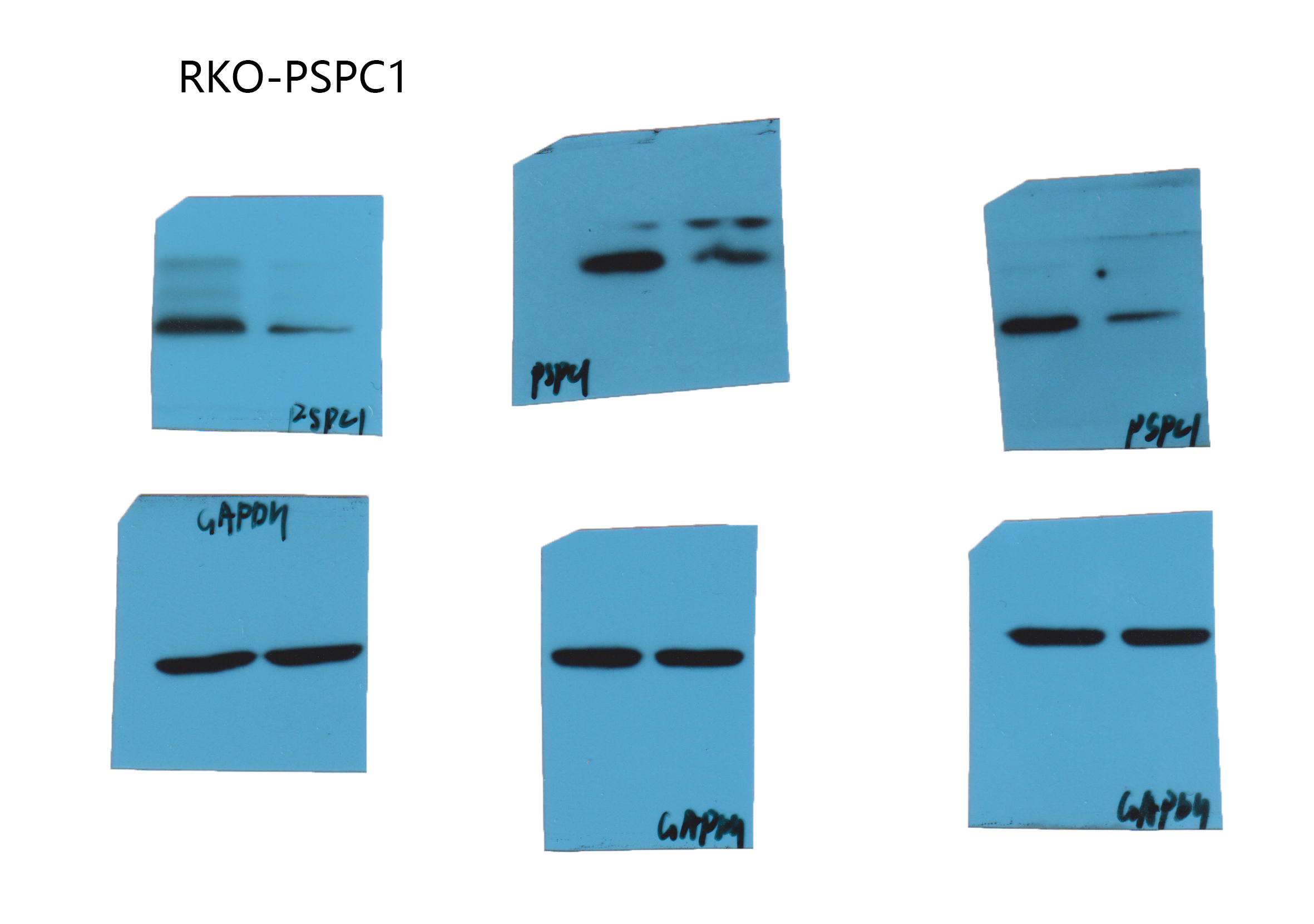

Supplement: Supplementary file 9 — Full and uncropped western blots [file 41420_2023_1384_MOESM9_ESM.tif]

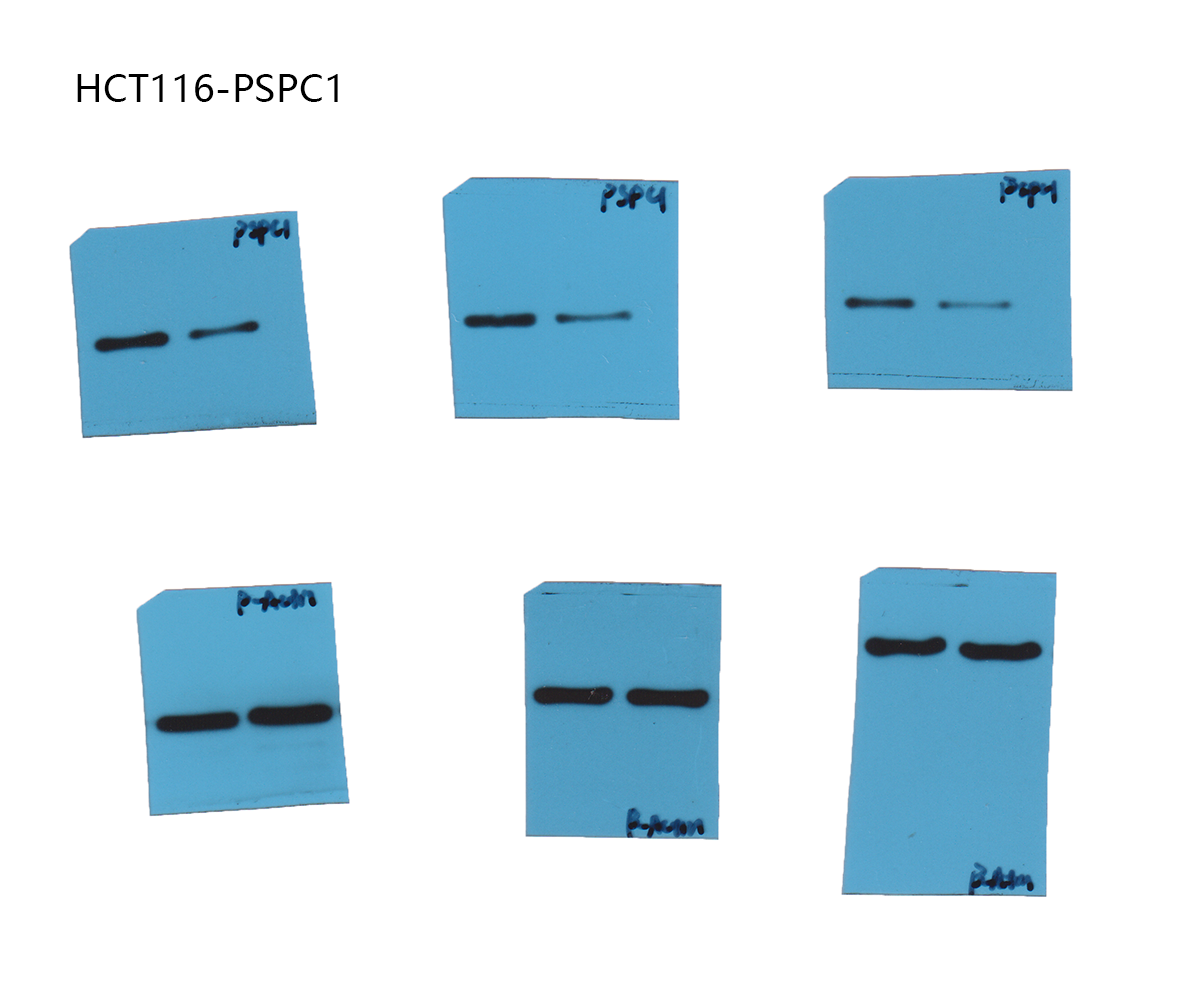

Supplement: Supplementary file 10 — Full and uncropped western blots [file 41420_2023_1384_MOESM10_ESM.tif]

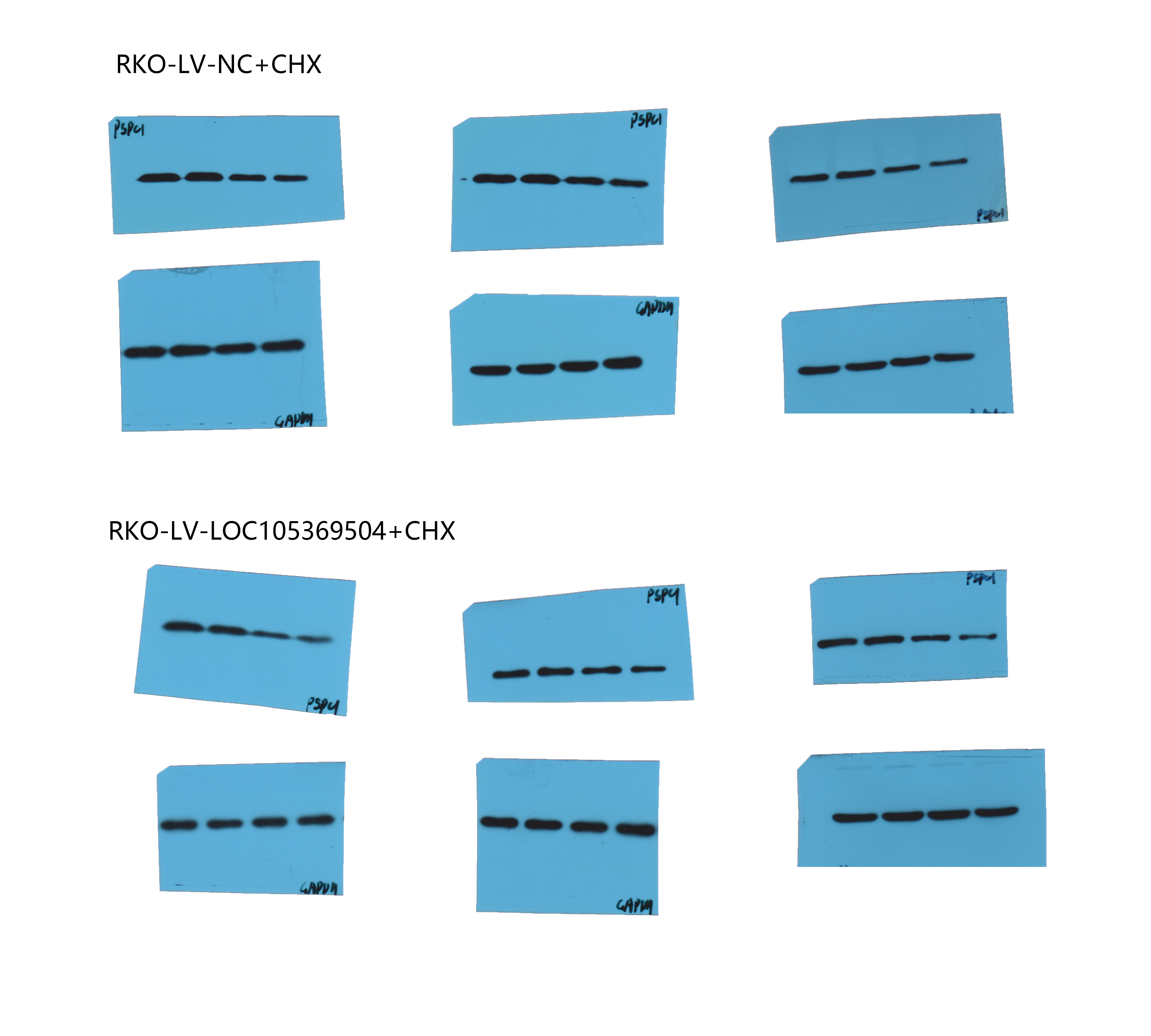

Supplement: Supplementary file 11 — Full and uncropped western blots [file 41420_2023_1384_MOESM11_ESM.tif]

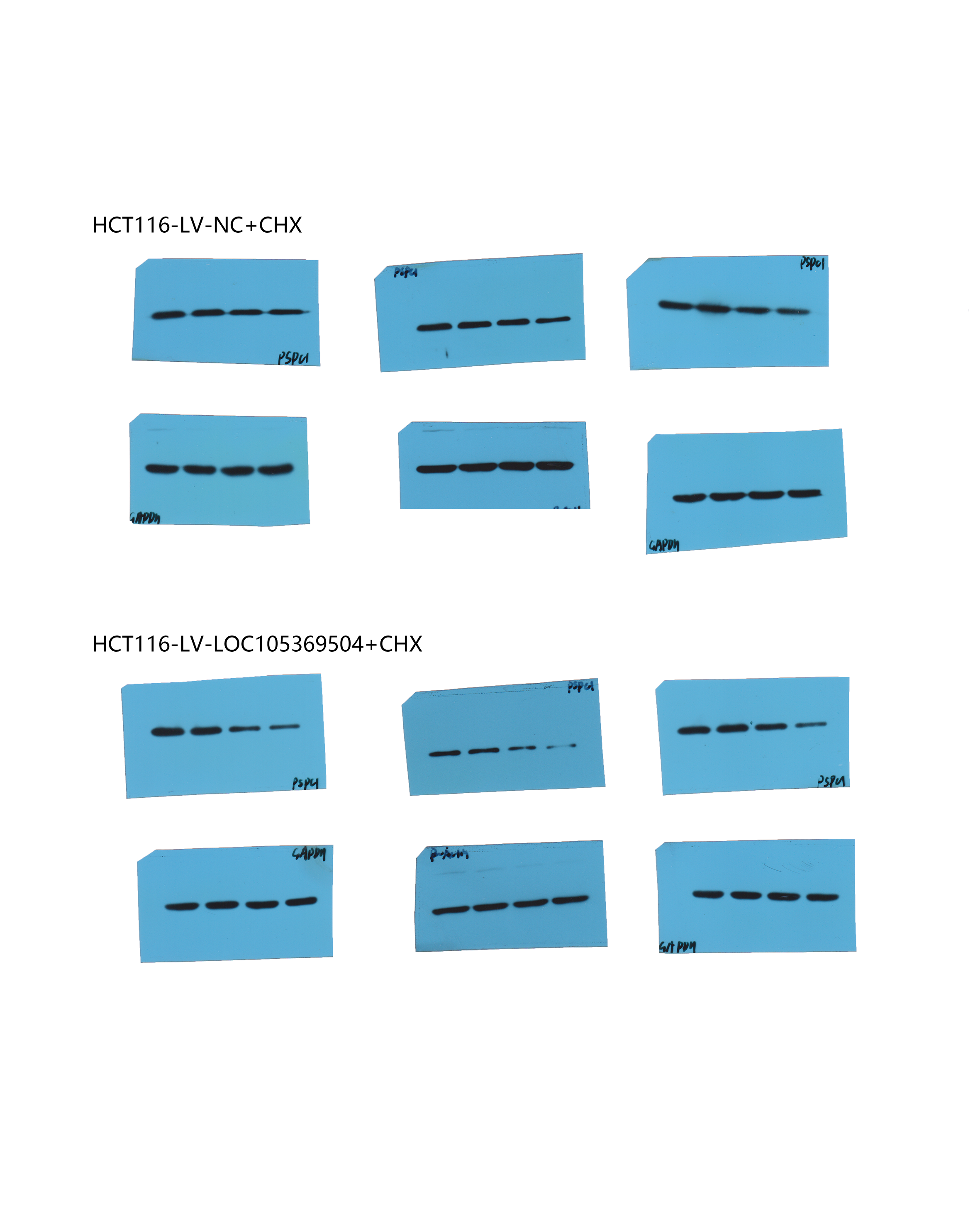

Supplement: Supplementary file 12 — Full and uncropped western blots [file 41420_2023_1384_MOESM12_ESM.tif]

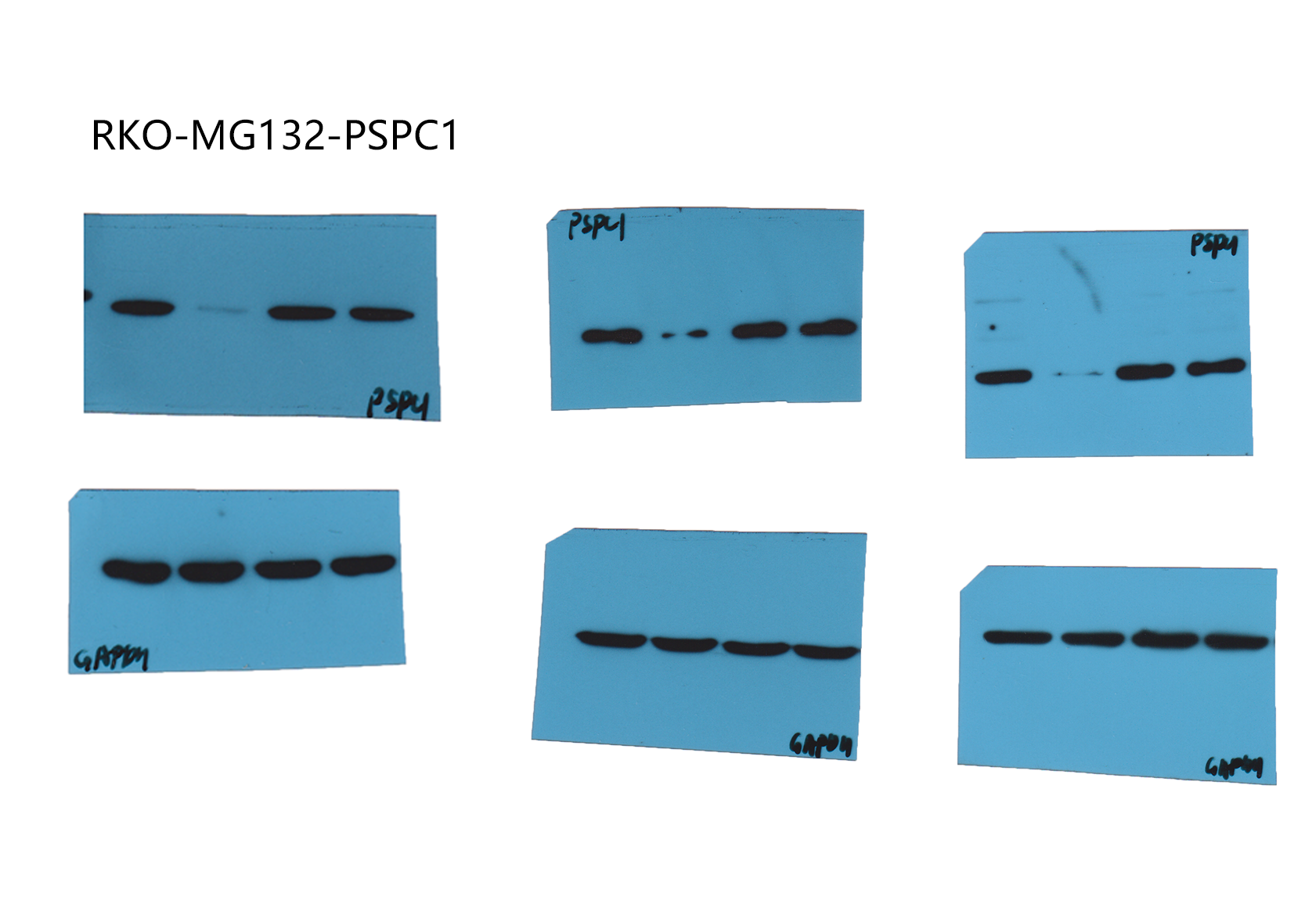

Supplement: Supplementary file 13 — Full and uncropped western blots [file 41420_2023_1384_MOESM13_ESM.tif]

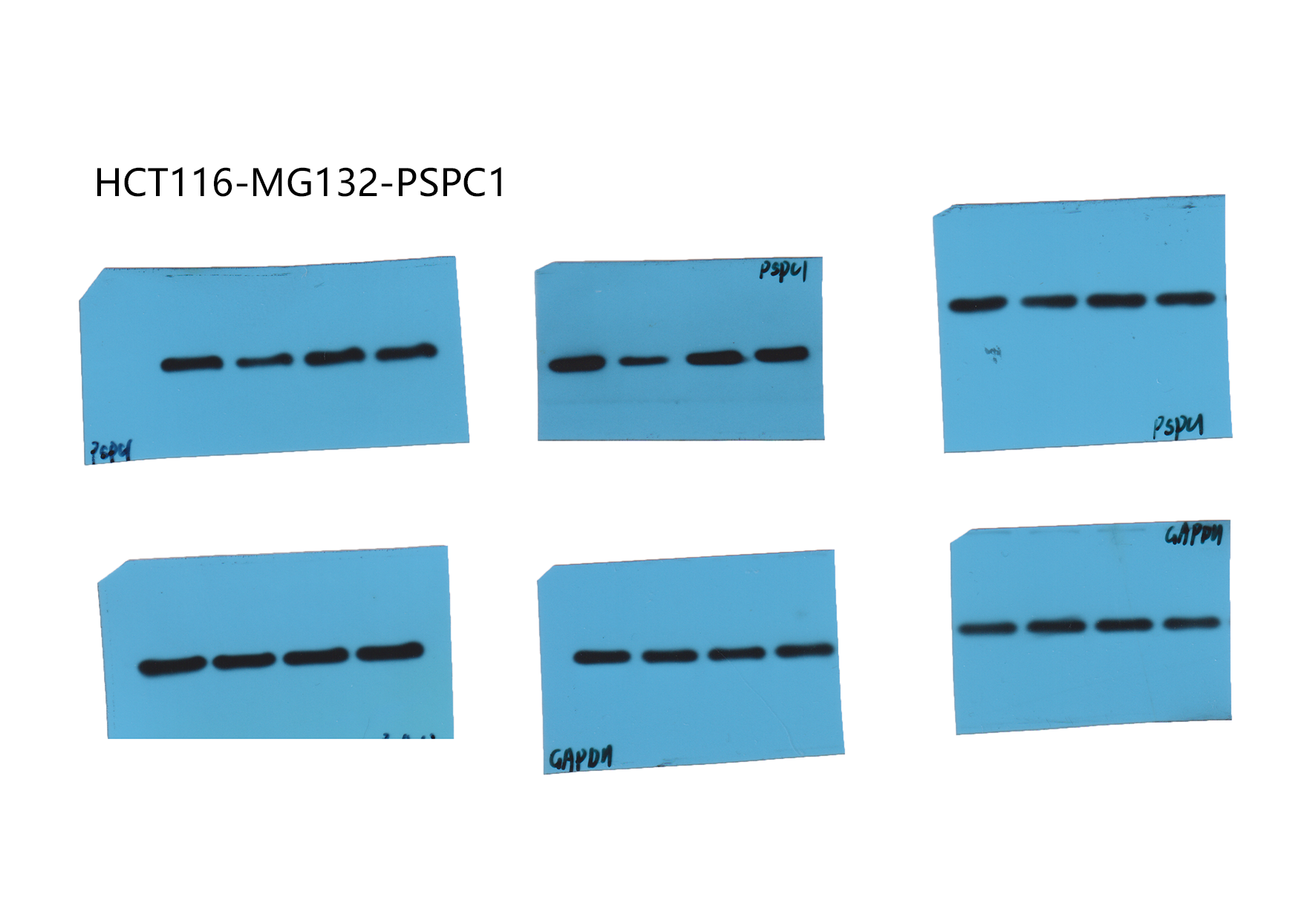

Supplement: Supplementary file 14 — Full and uncropped western blots [file 41420_2023_1384_MOESM14_ESM.tif]

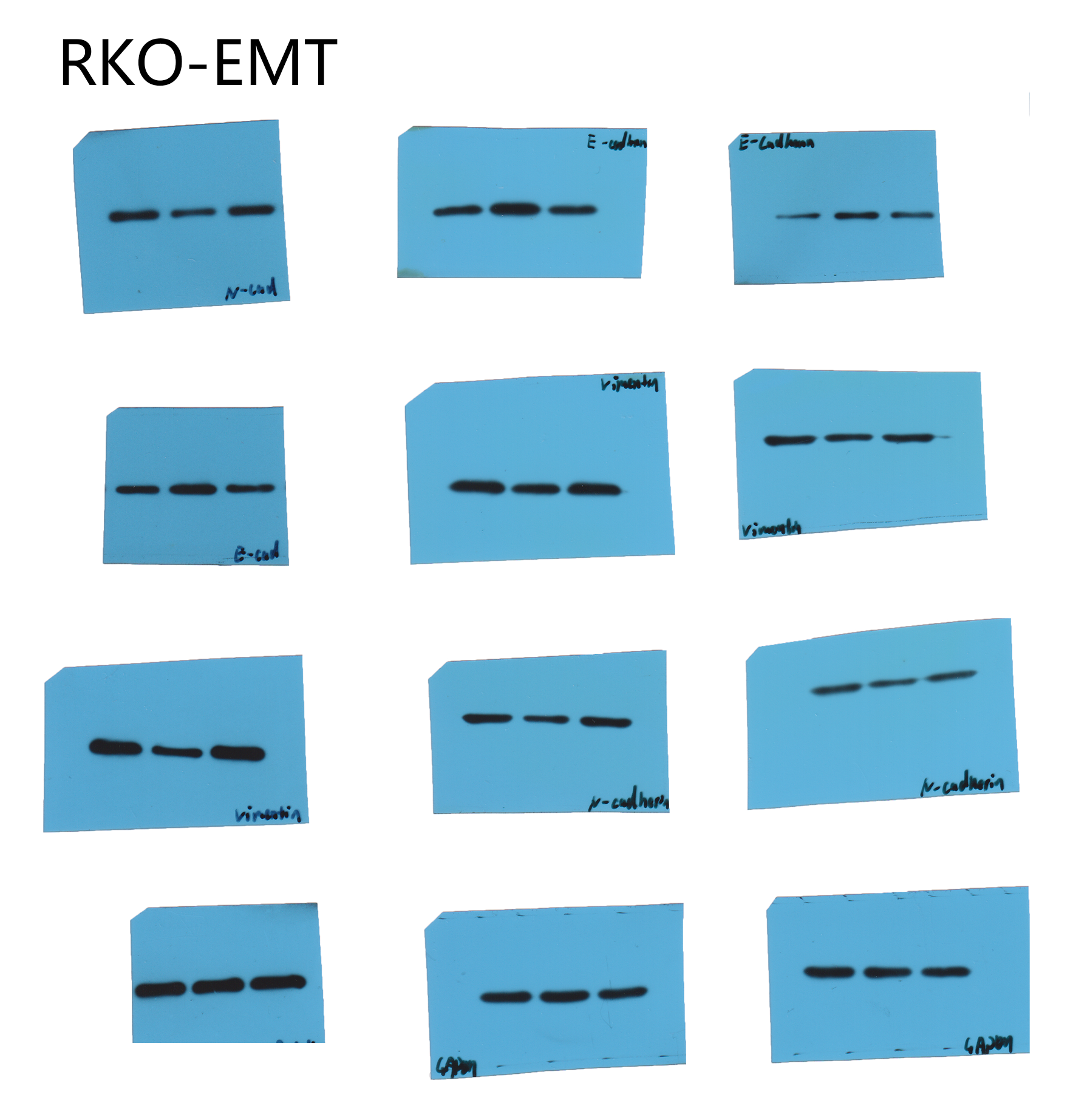

Supplement: Supplementary file 15 — Full and uncropped western blots [file 41420_2023_1384_MOESM15_ESM.tif]

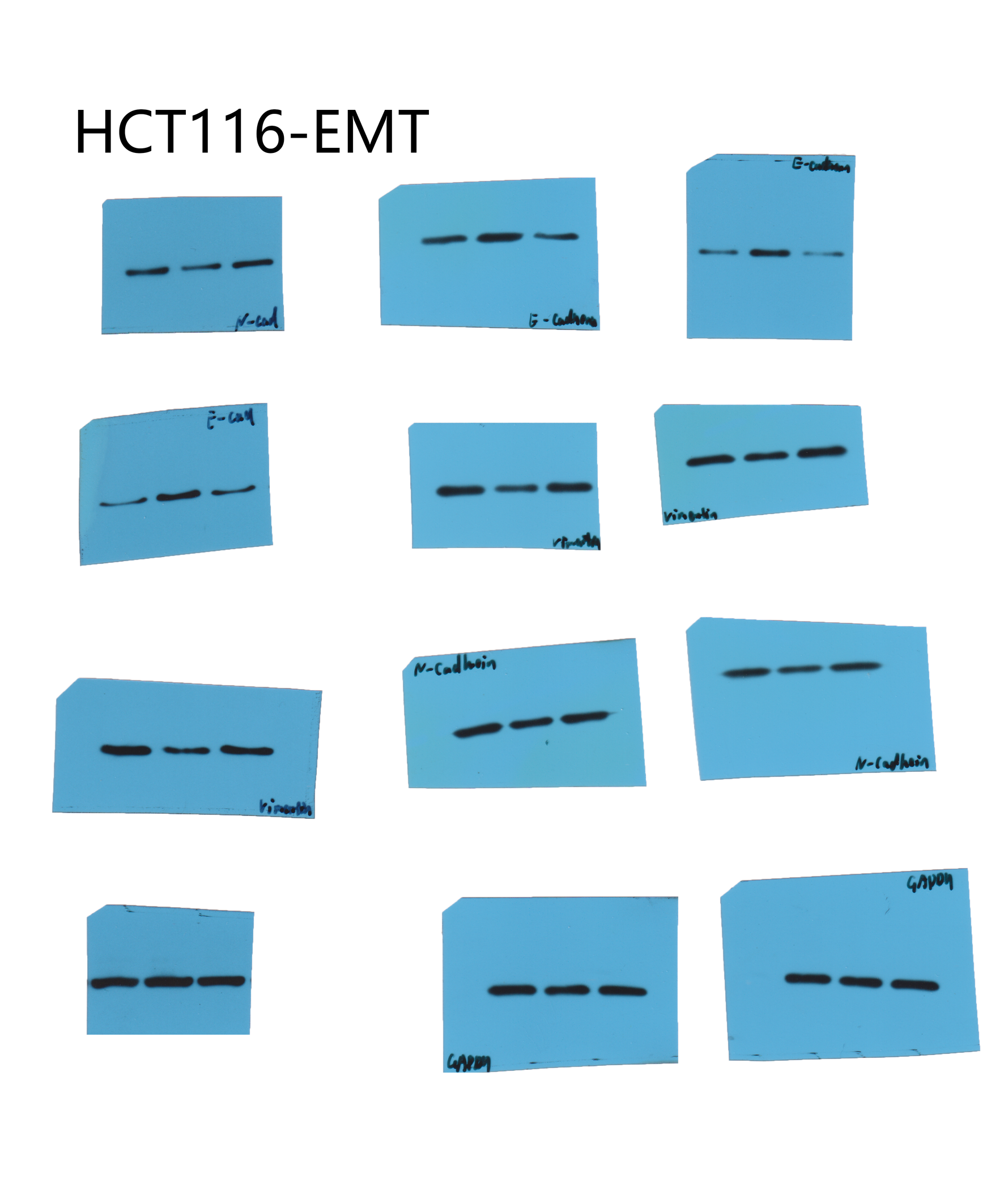

Supplement: Supplementary file 16 — Full and uncropped western blots [file 41420_2023_1384_MOESM16_ESM.tif]

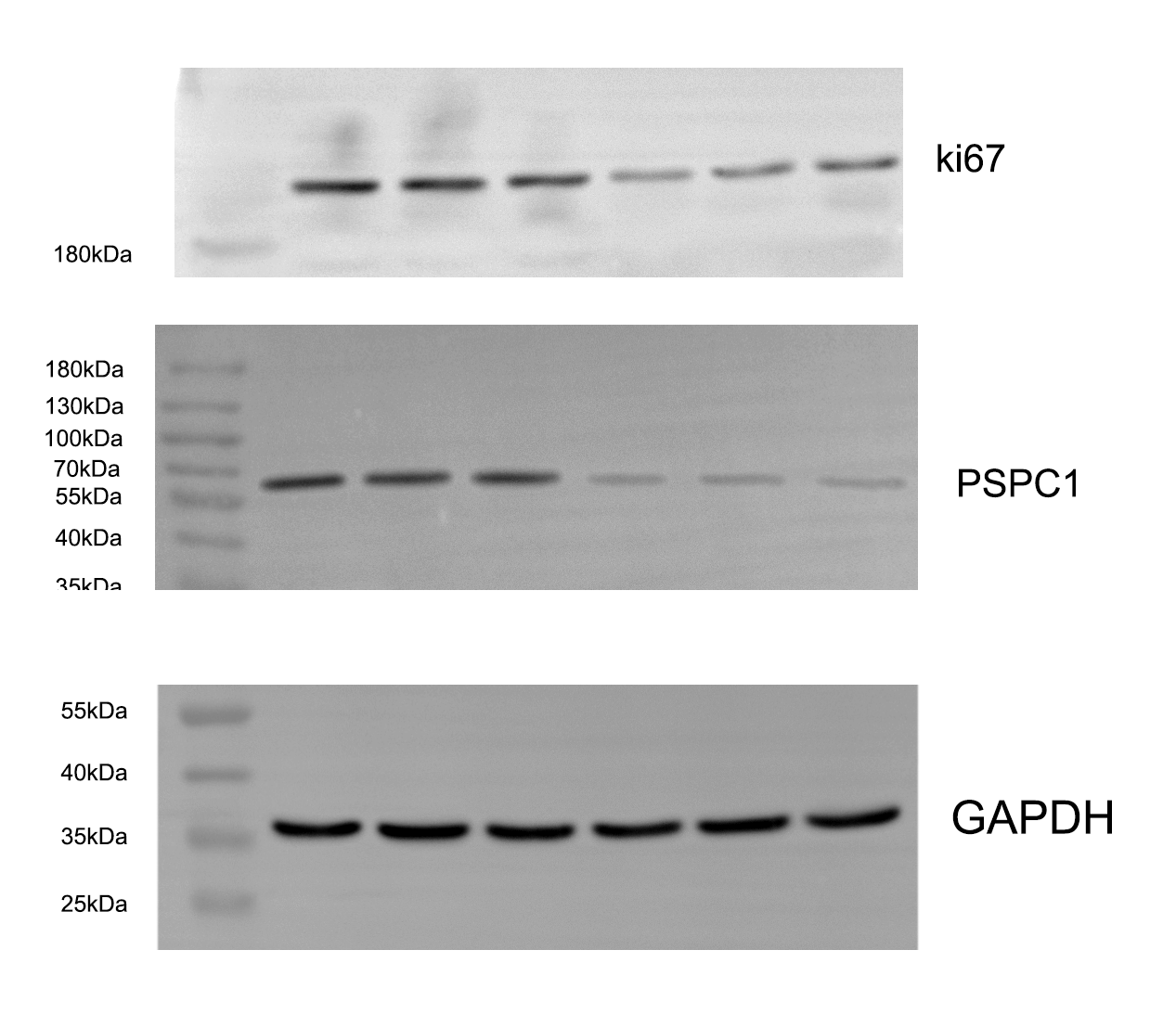

Supplement: Supplementary file 17 — Full and uncropped western blots [file 41420_2023_1384_MOESM17_ESM.tif]
