# Supplementary material for: LncRNA LOC105369504 inhibits tumor proliferation and metastasis in colorectal cancer by regulating PSPC1
Source: Cell Death Discov. 2023 Mar 10;9:89. doi: 10.1038/s41420-023-01384-3 (PMC9998613; doi:10.1038/s41420-023-01384-3)
Supplement: Supplementary file 3 — Table S1 [file 41420_2023_1384_MOESM3_ESM.docx]

**Table S1** Primers for real-time PCR.

| Primer name | Forward (5’-3’) | Reverse (5’-3’) |
| --- | --- | --- |
| LOC105369504 | AAAGTTCGCCTTTTCACATTCAG | CCCAAAAGATTCCCTATACCACATC |
| AL161431.1  18S  U6 | CGTTTTAATTCTGCCTCTCATTCTC  CTTAGTTGGTGGAGCGATTTG  CTCGCTTCGGCAGCACA | CCATCTTTCTCTATTGCCTGTGTG  GCTGAACGCCACTTGTCC  AACGCTTCACGAATTTGCGT |
| GAPDH | GAAGACGGGCGGAGAGAAAC | AAATGAGCCCCAGCCTTCTC |
