# Supplementary material for: LncRNA LOC105369504 inhibits tumor proliferation and metastasis in colorectal cancer by regulating PSPC1
Source: Cell Death Discov. 2023 Mar 10;9:89. doi: 10.1038/s41420-023-01384-3 (PMC9998613; doi:10.1038/s41420-023-01384-3)
Supplement: Supplementary file 5 — supplementary figure legends [file 41420_2023_1384_MOESM5_ESM.pdf]

Supplementary figure legends

Supplementary figure 1. The expression of LOC105369504 and lncRNA AL161431.1 decreased in CRC tissues compared with paracancerous tissues. Data are presented as the mean ± SD. *p < 0.05,***p < 0.001

Supplementary figure 2. The expression of LOC105369504 in LV-LOC105369504 overexpression cells was significantly up-regulated. Data are presented as the mean ± SD. *p < 0.05, **p < 0.01.

Table S1 . Primers for real-time PCR

Table S2. MS analysis showed that PSPC1 could bind to LOC105369504.
